# Smoking, Alcohol Drinking, Green Tea Consumption and the Risk of Esophageal Cancer in Japanese Men

**DOI:** 10.2188/jea.16.185

**Published:** 2006-09-04

**Authors:** Atsunobu Ishikawa, Shinichi Kuriyama, Yoshitaka Tsubono, Akira Fukao, Haruhiko Takahashi, Hidekiyo Tachiya, Ichiro Tsuji

**Affiliations:** 1 Division of Epidemiology, Department of Public Health and Forensic Medicine, Tohoku University Graduate School of Medicine.; 2 Tachiya Hospital.; 3 Division of Health Policy, Tohoku University School of Public Policy.; 4 Department of Public Health, Yamagata University School of Medicine.

**Keywords:** Alcohol Drinking, Esophageal Neoplasms, Tea, Risk, Smoking

## Abstract

**BACKGROUND:**

Although smoking and alcohol drinking are established risk factors of esophageal cancer, their public health impact is unclear. Furthermore, the effect of green tea is controversial.

**METHODS:**

The present study was based on a pooled analysis of two prospective cohort studies. A self-administered questionnaire about health habits was distributed to 9,008 men in Cohort 1 and 17,715 men in Cohort 2, aged 40 years or older, with no previous history of cancer. We identified 38 and 40 patient cases with esophageal cancer among the subjects in Cohort 1 (9.0 years of follow-up) and Cohort 2 (7.6 years of follow-up), respectively. Cox proportional hazards regression was used to estimate hazard ratios (HRs) of the risk of esophageal cancer incidence.

**RESULTS:**

Cigarette smoking, alcohol drinking and green tea consumption were significantly associated with an increased risk of esophageal cancer. Compared with men who had never smoked, never drunk alcohol or green tea, the pooled multivariate HRs (95% confidence intervals) were 5.09 (1.80-14.40) (p for trend <0.0001), 2.73 (1.55-4.81) (p for trend=0.0002), or 1.67 (0.89-3.16) (P for trend=0.04) for men who were currently smoking ≥20 cigarettes/day, drinking alcohol daily, or drinking ≥5 cups green tea/day, respectively. The population attributable fractions of esophageal cancer incidence that was attributable to smoking, alcohol drinking and green tea consumption were 72.0%, 48.6%, and 22.1%, respectively.

**CONCLUSIONS:**

Among the variables studied, smoking has the largest public health impact on esophageal cancer incidence in Japanese men, followed by alcohol drinking and green tea drinking.

Esophageal cancer is the sixth most common cause of cancer death among Japanese men. In 2004, 9,405 men died of esophageal cancer in Japan, accounting for about 5% of all cancer deaths.^1^

Although both smoking cigarettes and drinking alcohol have been consistently reported to be major risks for esophageal cancer,^2^^-^^18^ there might be other factors that increase or decrease the risk. One such factor may be green tea consumption. There have been conflicting data regarding the association between green tea consumption and cancer at various sites, such as the stomach,^19^^,^^20^ lung,^21^ breast,^22^^,^^23^ and colon.^24^ Green tea might have another type of influence on the incidence of esophageal cancer, and that is high temperature. Kinjo et al^4^ have indicated that drinking green tea at high temperature is associated with an increased risk of esophageal cancer mortality. However, their observation was based on a cohort study that started in 1965. Lifestyle-related factors of Japanese people, including green tea consumption, have been changing drastically over the last few decades.

In areas where cigarette smoking, alcohol drinking, and green tea consumption are widespread, the relationship between the use of these three substances and esophageal cancer requires epidemiologic studies. The population attributable fraction (PAF), which is defined as the proportion of disease in the population that is attributable to a given risk factor, is useful for estimating the public health impact of the factor. However, only two studies from the USA and Taiwan have examined PAFs of esophageal cancer using a case-control design.^25^^,^^26^ To our knowledge, no such data are available for Japan.

The purpose of the present study was to investigate the risk of esophageal cancer incidence associated with cigarette smoking, alcohol drinking, and green tea consumption in a population-based prospective cohort and to calculate the PAF for each factor if it was significantly associated with such a risk.

## METHODS

### Study cohort

The present study was based on a pooled analysis of two prospective cohort studies conducted in Miyagi Prefecture, Japan. Details of the design for each study have been reported elsewhere.^19^^,^^20^^,^^22^^,^^24^^,^^27^^-^^29^ For cohort 1, we delivered a self-administered questionnaire in January 1984 to all residents aged 40 years or older (n=33,453) in 3 municipalities of Miyagi Prefecture. Usable questionnaires were returned from 31,345 (93.7%; 13,991 men and 17,354 women) of the subjects. For cohort 2, we delivered a self-administered questionnaire between June and August 1990 to all residents aged 40-64 years (n=51,921) in 14 municipalities of Miyagi Prefecture. Usable questionnaires were returned from 47,605 (91.7%; 22,836 men and 24,769 women) of the subjects. The study protocol was approved by the institutional review board of Tohoku University School of Medicine. We considered the return of the self-administered questionnaires signed by the subjects to imply their consent to participate in the study.

Because women in these cohorts seldom smoked cigarettes or drank alcohol, we limited our analysis to men (n=13,991 for cohort 1; 22,836 for cohort 2). We excluded subjects who already had cancer at the time of the baseline survey (258 subjects in cohort 1 and 424 subjects in cohort 2). We then excluded 9,422 subjects (4,725 for cohort 1; 4,697 for cohort 2) because they did not answer the question on smoking, alcohol drinking, or green tea consumption. Consequently, our analysis included 9,008 men including 38 cases of esophageal cancer in cohort 1, and 17,715 men including 40 cases of esophageal cancer in cohort 2.

### Exposure data

In both cohorts, the questionnaire included items related to smoking, alcohol drinking, frequency of green tea consumption, and food items consumed.

For cigarette smoking, we classified the subjects into four categories: ‘never smoked’, ‘formerly smoked’, ‘currently smoking 1-19 cigarettes/day’, and ‘currently smoking ≥20 cigarettes/day’.

For alcohol drinking, we classified the subjects into three categories: ‘never or occasionally drank’, ‘formerly drank’, and ‘daily drinking’. In this analysis, we defined ‘daily drinking’ as those drinking alcohol 5 days or more per week. We also defined ‘occasionally drank’ as those who reported drinking alcohol less than 5 days per week. We did not consider the types or quantity of alcohol beverages they consumed.

For green tea consumption, we classified the subjects into four categories: ‘never drank’, ‘drinking 1-2 cups/day’, ‘drinking 3-4 cups/day’, and ‘drinking ≥5 cups/day’.

### Follow-up

The end point in this study was the incidence of esophageal cancer defined as topography code C15.0-C15.9 according to the International Classification of Diseases for Oncology (2^nd^ Ed.; ICD-O-2).^30^

For both cohorts, we followed the vital and residential status of each study subject with a population registry maintained by each municipality. We ascertained the incidence of esophageal cancer during the follow-up with a linkage to records kept at Miyagi Prefectural Cancer Registry. A follow-up was conducted from January 1, 1984 through December 31, 1992 for cohort 1, and from June 1, 1990 through December 31, 1997 for cohort 2.

### Statistical analysis

We used Cox proportional-hazards regression to estimate the hazard ratios (HRs) and 95% confidence intervals (CIs) of esophageal cancer incidence according to categories of smoking cigarettes, drinking alcohol, and drinking green tea, and to adjust for potentially confounding variables, using the PHREG procedure on the SAS^®^ version 9.1 statistical software package (SAS Inc., Cary, NC, USA).

We considered the following variables to be potential confounding factors: age in years, cigarette smoking (never, past, current smoking 1-19 cigarettes/day, or current smoking ≥20 cigarettes/day; when calculating the HRs and their 95% CIs for alcohol drinking or green tea consumption), alcohol drinking (never and occasionally, former, daily; when calculating the HRs and their 95% CIs for smoking or green tea consumption), green tea consumption (never, 1-2 cups/day, 3-4 cups/day, ≥5 cups/day; when calculating the HRs and their 95% CIs for smoking or alcohol drinking), coffee consumption (never or occasionally, 1-2 cups/day, ≥3 cups/day), and black tea consumption (never or occasionally, ≥1 cup/day).

Interactions among the variables for smoking, alcohol drinking, and green tea consumption were assessed through addition of cross-product terms to the multivariate model. We performed additional analysis to investigate the joint effects of these variables by estimating HRs in combined categories of the variables. For this analysis, the categories of smoking, alcohol drinking, and green tea consumption were divided into currently smoking or other, currently alcohol drinking or other, and currently consuming ≥3 cups/day green tea or other, respectively.

Because smoking, alcohol drinking and green tea consumption are modifiable risk factors, we calculated the PAF, an estimate of the proportion of esophageal cancer incidence in Japan that might be avoided if the population were not exposed to these risk factors. PAF was calculated as

*pd* × (HR − 1)/HR,

where *pd* is the proportion of cases exposed to the risk factor.^31^

To obtain a summary measure of the results from cohort 1 and cohort 2, we used the general variance-based method.^32^ All p-values are two-tailed, and differences at p<0.05 were considered to be statistically significant.

## RESULTS

Table 1 compares the characteristics of subjects according to smoking, alcohol drinking, and green tea consumption. The subjects in cohort 1 who were heavy smokers (20 cigarettes or more/day) tended to be younger and were more likely to be daily alcohol drinkers and higher green tea consumers. We observed a similar tendency in cohort 2 among heavy smokers. The characteristics of the subjects who were daily drinkers differed between the two cohorts. The subjects who were higher green tea consumers tended to be older both in cohort 1 and cohort 2.

**Table 1.** Baseline characteristics of the study subjects according to smoking, alcohol drinking, and green tea consumption categories.

	Characteristics of cohort 1							Characteristics of cohort 2					
	
	Smoking			Alcohol drinking		Green tea consumption		Smoking		Alcohol drinking		Green tea consumption	
					
	Never		Current smoking ≥20 cigarettes/day	Never or occasionally	Daily	Never or occasionally	≥5 cups/day	Never	Current smoking ≥20 cigarettes/day	Never or occasionally	Daily	Never or occasionally	≥5 cups/day
No. of subjects	2007 (100)		3270 (100)	4029 (100)	4244 (100)	1663 (100)	3657 (100)	3498 (100)	7855 (100)	7743 (100)	8728 (100)	5200 (100)	4426 (100)

Age (years) [standard deviation]	56.7 [12.4]		53.1 [9.3]	56.9 [11.4]	54.0 [10.0]	55.6 [11.4]	56.9 [10.7]	51.4 [7.1]	50.1 [7.5]	50.9 [7.6]	51.1 [7.5]	50.3 [7.5]	53.5 [7.4]

Smoking, No. of subjects (%)*													
Former	-		-	925 (23.0)	874 (20.6)	393 (23.6)	843 (23.1)	-	-	1508 (19.5)	1751 (20.1)	1044 (21.1)	916 (20.7)
Current smoking <20 cigarettes/day	-		-	628 (15.6)	854 (20.1)	323 (19.4)	638 (17.5)	-	-	1122 (14.5)	1355 (15.5)	838 (16.1)	594 (13.4)
Current smoking ≥20 cigarettes/day	-		-	1242 (30.8)	1868 (44.0)	497 (29.9)	1500 (41.0)	-	-	3035 (39.2)	4375 (50.1)	2219 (42.7)	2154 (48.7)

Alcohol drinking, No. of subjects (%)*													
Former	125 (6.2)		160 (4.9)	-	-	176 (10.6)	279 (7.6)	173 (5.0)	445 (5.7)	-	-	386 (7.4)	355 (8.0)
Daily	648 (32.3)		1868 (57.1)	-	-	752 (45.2)	1693 (46.3)	1247 (35.7)	4375 (55.7)	-	-	2556 (49.2)	2057 (46.5)

Green tea consumption, No. of subjects (%)*													
1-2 cups/day	384 (19.1)		595 (18.2)	709 (17.6)	813 (19.2)	-	-	869 (24.8)	1856 (23.6)	1798 (23.2)	2212 (25.3)	-	-
3-4 cups/day	497 (24.8)		678 (20.7)	900 (22.3)	986 (23.2)	-	-	768 (22.0)	1626 (20.7)	1673 (21.6)	1903 (21.8)	-	-
≥5 cups/day	676 (33.7)		1500 (45.9)	1685 (41.8)	1693 (39.9)	-	-	762 (21.8)	2154 (27.4)	2014 (26.0)	2057 (23.6)	-	-

Coffee consumption, No. of subjects (%)*													
1-2 cups/day	355 (17.7)		877 (26.8)	942 (23.4)	997 (23.5)	302 (18.2)	794 (21.7)	874 (25.0)	2380 (30.3)	2280 (29.5)	2352 (27.0)	1460 (28.1)	956 (21.6)
≥3 cups/day	107 (5.3)		490 (15.0)	437 (10.9)	344 (8.1)	165 (9.9)	301 (8.2)	293 (8.4)	1683 (21.4)	1297 (16.8)	113 (13.0)	890 (17.1)	539 (12.2)

Black tea consumption, No. of subjects (%)*													
≥1 cup/day	123 (6.1)		142 (4.3)	235 (5.8)	199 (4.7)	46 (2.8)	180 (4.9)	80 (2.3)	180 (2.3)	191 (2.5)	193 (2.2)	88 (1.7)	112 (2.5)

*: The percentage was calculated by dividing the number of subjects in the cell by total number of subjects in the column.

Table 2 shows the association between smoking, alcohol drinking, green tea consumption and the risk of esophageal cancer. We found that cigarette smoking, alcohol drinking, and green tea consumption were significantly associated with an increased risk of esophageal cancer. The pooled multivariate HR (95% CI) for esophageal cancer in subjects who never smoked, formerly smoked, currently smoking 1-19 cigarettes/day, and currently smoking ≥20 cigarettes/day were 1.00, 2.07 (0.66-6.57), 5.00 (1.70-14.7) and 5.09 (1.80-14.4), respectively (p for trend <0.0001). Analysis of each cohort demonstrated a similar trend. In comparison with smoking, the impact of alcohol drinking on esophageal cancer risk was relatively moderate, but the risk among current drinkers was 2.7 times higher than that among the non-drinkers.

**Table 2.** Hazard ratios (HRs) and their confidence intervals (CIs) for esophageal cancer risk according to smoking, alcohol drinking and green tea consumption categories.

	Category				P for trend
	Smoking				

	Never	Former	Current smoking 1-19 cigarettes per day	Current smoking ≥20 cigarettes per day	
Cohort 1					
Cases/person-years	2/15277.9	6/15385.8	11/11996.4	19/24415.3	
Age-adjusted HR (95% CI)	1.00	2.88 (0.58-14.27)	7.07 (1.57-31.90)	7.42 (1.71-32.19)	0.001
Multivariate HR (95% CI)*	1.00	2.49 (0.50-12.44)	5.39 (1.18-24.61)	5.48 (1.24-24.18)	0.008

Cohort 2					
Cases/person-years	2/25768.2	5/26942.7	10/19511.4	23/57389.1	
Age-adjusted HR (95% CI)	1.00	1.87 (0.36-9.66)	5.29 (1.16-24.22)	5.72 (1.35-24.24)	0.002
Multivariate HR (95% CI)*	1.00	1.72 (0.33-8.92)	4.63 (1.01-21.30)	4.73 (1.10-20.34)	0.006

Pooled multivariate HR (95% CI)*	1.00	2.07 (0.66-6.57)	5.00 (1.70-14.66)	5.09 (1.80-14.40)	<0.0001
	Alcohol drinking				

	Never or occasionally	Former	Daily		

Cohort 1					
Cases/person-years	8/30445.4	4/4890.0	26/31740.0		
Age-adjusted HR (95% CI)	1.00	2.57 (0.77-8.61)	3.53 (1.59-7.85)		0.002
Multivariate HR (95% CI)*	1.00	2.31 (0.68-7.83)	2.70 (1.20-6.06)		0.017

Cohort 2					
Cases/person-years	8/56756.3	1/8914.2	31/63940.8		
Age-adjusted HR (95% CI)	1.00	0.54 (0.07-4.30)	3.45 (1.58-7.51)		0.001
Multivariate HR (95% CI)*	1.00	0.50 (0.06-4.04)	2.77 (1.26-6.09)		0.007

Pooled multivariate HR (95% CI)*	1.00	1.55 (0.58-4.14)	2.73 (1.55-4.81)		0.0002
	Green tea consumption				

	Never or occasionally	1-2 cups/day	3-4 cups/day	≥5 cups/day	
Cohort 1					
Cases/person-years	5/12514.9	3/11910.9	9/14921.3	21/27728.3	
Age-adjusted HR (95% CI)	1.00	0.64 (0.15-2.66)	1.45 (0.49-4.34)	1.78 (0.67-4.72)	0.09
Multivariate HR (95% CI)*	1.00	0.69 (0.17-2.91)	1.58 (0.52-4.76)	1.78 (0.66-4.82)	0.11

Cohort 2					
Cases/person-years	9/38004.7	8/31226.0	6/27962.4	17/32418.3	
Age-adjusted HR (95% CI)	1.00	1.14 (0.44-2.94)	0.78 (0.28-2.18)	1.49 (0.66-3.37)	0.39
Multivariate HR (95% CI)*	1.00	1.22 (0.47-3.19)	0.85 (0.30-2.40)	1.61 (0.71-3.66)	0.31

Pooled multivariate HR (95% CI)*	1.00	1.03 (0.46-2.28)	1.13 (0.53-2.42)	1.67 (0.89-3.16)	0.04

* : Adjusted for age in years, cigarette smoking (never, past, current smoking 1-19 cigarettes/day, or current smoking ≥20 cigarettes/day; when calculating the HRs and their 95% CIs for alcohol drinking or green tea consumption), alcohol drinking (never and occasionally, former, daily; when calculating the HRs and their 95% CIs for smoking or green tea consumption), green tea consumption (never, 1-2 cups/day, 3-4 cups/day, ≥5 cups/day; when calculating the HRs and their 95% CIs for smoking or alcohol drinking), coffee consumption (never or occasionally, 1-2 cups/day, ≥3 cups/day), and black tea consumption (never or occasionally, ≥1 cup/day).

As compared with subjects who never drank green tea, the incidence risk of esophageal cancer was increased among those drinking 1-2 cups/day (HR=1.03, 95% CI=0.46-2.28), those drinking 3-4 cups/day (HR=1.13, 95% CI=0.53-2.42) and those drinking 5 cups or more/day (HR=1.67, 95% CI=0.89-3.16). In contrast to the higher risk for smoking and alcohol drinking, the risk for green tea was modest. However, we observed a significant dose-response relationship (p for trend=0.04).

We further examined the relationship between the risk of esophageal cancer and the consumption of coffee, but found no association. After adjustment for age, cigarette smoking, alcohol drinking, green tea consumption, and black tea consumption, the pooled multivariate HRs (95% CIs) for esophageal cancer in subjects who were drinking 1-2 cups/day, or drinking 3 cups or more/day were 0.63 (0.32-1.27) and 0.94 (0.36-2.45), respectively, compared with subjects who never drank coffee (p for trend=0.41).

None of the tests for interactions among the variables of smoking, alcohol drinking, and green tea consumption showed significant results. However, we observed potential effect modifications by analysis of combined categories of these variables. Table 3 lists the joint effects of smoking, alcohol drinking, and green tea consumption on the risk of esophageal cancer. The comparison showed that the HRs were very high when smoking and alcohol drinking were present simultaneously.

**Table 3.** Hazard ratios (HRs) and their 95% confidence intervals (95% CIs) for esophageal cancer risk according to combined categories of smoking, alcohol drinking and green tea consumption.

Smoking*	Alcohol^†^	Green tea^‡^	Cohort 1		Cohort 2		
	
			No. of cases/ person-years	HR (95% CI)^§^	No. of cases/ person-years	HR (95% CI)^§^	Pooled
							HR (95% CI)^§^
−	−	−	1 / 7508.1	1.00 (referent)	1 / 15933.7	1.00 (referent)	1.00 (referent)
＋	−	−	0 / 5208.6	-	2 / 18334.4	2.30 (0.21-25.5)	-
−	＋	−	2 / 4351.8	4.40 (0.40-48.8)	1 / 12685.7	1.31 (0.08-20.9)	2.61 (0.42-16.07)
−	−	＋	4 / 11729.7	2.47 (0.28-22.2)	1 / 14726.1	0.86 (0.05-13.7)	1.65 (0.29- 9.19)
＋	＋	−	5 / 7357.4	7.25 (0.83-63.0)	13 / 22276.9	11.4 (1.49-87.3)	9.23 (2.10-40.60)
＋	−	＋	7 / 10889.1	5.65 (0.69-46.2)	5 / 16676.3	4.39 (0.51-37.6)	4.99 (1.11-22.43)
−	＋	＋	1 / 7074.2	1.21 (0.08-19.4)	4 / 9365.4	5.20 (0.58-46.6)	2.97 (0.53-16.58)
＋	＋	＋	18 / 12956.6	12.2 (1.61-92.5)	13 / 19612.9	10.0 (1.31-76.7)	11.1 (2.63-46.51)

* : Currently smoking

† : Daily alcohol drinking

‡ : Daily consumption of ≥3 cups/day

§ : Adjusted for age in years, coffee consumption (never or occasionally, 1-2 cups/day, ≥3 cups/day), and black tea consumption (never or occasionally, ≥1 cup/day).

The proportion of esophageal cancer incidence attributable to smoking or alcohol drinking was very high. The PAFs of esophageal cancer incidence attributable to smoking and alcohol drinking were 72.0% and 48.6%, respectively (Table 4), whereas that attributable to green tea consumption was 22.1% (Table 4).

**Table 4.** Population attributable fraction according to smoking, alcohol drinking or green tea consumption for esophageal cancer risk in Japanese men.

Smoking				Alcohol drinking				Green tea			
		
	Prevalence of cases (%)	Hazard ratio*	Population attributable fraction (%)^†^		Prevalence of cases (%)	Hazard ratio*	Population attributable fraction (%)^†^		Prevalence of cases (%)	Hazard ratio*	Population attributable fraction (%)^†^
Former	14.1	2.07	7.3	Former	6.4	1.55	2.3	1-2 cups/day	14.1	1.03	0.4
Current smoking<20 cigarettes/day	26.9	5.00	21.5	Daily	73.1	2.73	46.3	3-4 cups/day	19.2	1.13	2.2
Current smoking≥20 cigarettes/day	53.8	5.09	43.2					≥5 cups/day	48.7	1.67	19.5

Total population attributable fraction			72.0				48.6				22.1

* : Data on hazard ratios were from the results of the present study.

† : The population attributable fractions were calculated with the use of Equation 5 in Rockhill et al.^31^

## DISCUSSION

In this pooled analysis of two prospective cohorts, we found a significant positive association between smoking, alcohol drinking, green tea consumption and increased risk of esophageal cancer. The HRs were very high when smoking and alcohol drinking were present simultaneously. The PAFs of incident esophageal cancer in this population that were attributable to smoking, alcohol drinking, or green tea consumption were 72.0%, 48.6%, or 22.1%, respectively. To our knowledge, this is the first study to report the PAFs for esophageal cancer from Japan.

Our results for smoking and alcohol drinking are consistent with those of previous studies.^2^^-^^18^ Furthermore, the joint effect of these variables is consistent with previous reports.^4^^,^^5^^,^^26^^,^^33^^-^^35^ At least more than additive synergistic effects were shown in four case-control studies^5^^,^^26^^,^^33^^,^^35^ and two prospective cohort studies.^4^^,^^34^ We also observed very high HRs when smoking and alcohol drinking were present simultaneously.

Although studies using laboratory animals have suggested inhibitory effects of green tea on the induction of esophageal cancer,^36^ only a few studies have evaluated the relationship in humans, and the results were substantially conflicting.^4^^,^^37^ Gao et al^37^ observed a protective effect of green tea drinking on esophageal cancer incidence among women (odds ratio=0.50; 95% CI=0.30-0.83) but not among men using a case-control study design among Chinese subjects. In contrast, Kinjo et al^4^ demonstrated that rate ratio and 95% CI were 1.6 (1.2-2.0) for hot tea (drinking green tea at high temperature) in comparison with non-hot tea (drinking green tea at moderate temperature) in a prospective cohort study among Japanese subjects. The results obtained by Gao et al^37^ may have been affected by recall bias. Using a prospective study design, we observed that green tea consumption was associated with an increased risk, which was consistent with the results of Kinjo et al^4^ One plausible explanation for our result was the effect of high tea temperature, although we had no information about the temperature of the green tea consumed. However, coffee, which is also generally consumed at high temperature in Japan, was not associated with an increased risk in our study. Therefore, a mechanism other than high temperature may be considered. Our findings for green tea require confirmation by future studies because prospective data on this relationship are scarce.

With respect to the PAFs of smoking and alcohol drinking, only two reports are available to date.^25^^,^^26^ Engel et al^25^ from the United States demonstrated that PAFs for ever smoking and any alcohol consumption for esophageal carcinoma in men were 57.6% and 80.2%, respectively. The corresponding figures by Lee et al^26^ from Taiwan were 63.4% and 66.9%, respectively. In contrast to the previous studies, the PAF for incident esophageal cancer was larger in smokers than in alcohol drinkers in our study. The high PAF in smokers was due to both the higher prevalence of smoking and HR in smokers than in alcohol drinkers. Among the subjects, 57.6% (54.1% for cohort 1 and 59.4% for cohort 2) were current smokers and 48.5% (47.1% for cohort 1 and 49.3% for cohort 2) were daily drinkers. The pooled multivariate HR (95% CI) for esophageal cancer in subjects who had never smoked, formerly smoked, were currently smoking 1-19 cigarettes/day, and currently smoking ≥ 20 cigarettes/day were 1.00, 2.07 (0.66-6.57), 5.00 (1.70-14.66), and 5.09 (1.80-14.40), respectively, while the corresponding values in subjects who never or only occasionally drank, former drinkers, and daily drinkers were 1.00, 1.55 (0.58-4.14), and 2.73 (1.55-4.81), respectively. Our data suggest that cessation and/or primary prevention of smoking have priority over the alcohol issue for reducing esophageal cancer in a population-based manner.

Our study had several strengths. We recruited our subjects from the general population. The information on exposures was obtained before the cases of esophageal cancer had been diagnosed, thus avoiding any effect of recall bias. In addition, the positive association between smoking, alcohol drinking, green tea consumption and the risk of esophageal cancer was unchanged after adjustment for potential confounders.

Our study also had some limitations. First, our sample size of 78 cases of esophageal cancer (38 cases for cohort 1 and 40 cases for cohort 2) may not have been sufficient for analyzing the joint effects of the variables, because these analyses were conducted with a higher number of categories than independent analysis. If we had obtained more samples, we might have been able to observe significant results from the interaction tests.

Second, because information on exposures was based on self-administered questionnaires and was collected once only, some misclassification of subjects was inevitable. Nevertheless, because the information was collected before subjects developed esophageal cancer or other serious diseases, any misclassification of exposures would likely have been non-differential and resulted in conservative estimates for the association between smoking, alcohol drinking, green tea consumption and the risk of esophageal cancer.

Third, we excluded 9,422 subjects (4,725 for cohort 1; 4,697 for cohort 2) because they did not answer the question on smoking, alcohol drinking, or green tea consumption. Forty-one cases (26 in cohort 1 and 15 in cohort 2) of esophageal cancer were diagnosed in this group. We considered that the characteristics of subjects who did not report these exposures were essentially similar to those of subjects who did, as there was no difference in mean age (58.2 and 56.0, respectively) and the age-adjusted HR for esophageal cancer in the subjects who did not answer the question about their smoking, drinking, or green tea consumption status, as compared to those who did, was not significant (1.22, 95% CI; 0.70-2.01) in cohort 1. Similar results, for both characteristics and HR, were obtained in cohort 2. Corresponding mean ages were 51.3 and 53.6, respectively, and corresponding age-adjusted HR (95% CI) was 1.10 (0.61-1.99). Thus, our results would not be substantially biased by exclusion of the subjects who did not answer the questions on exposures.

In conclusion, we have found that smoking, alcohol drinking or green tea consumption is significantly associated with an increased incidence risk of esophageal cancer. To reduce esophageal cancer risk, primary prevention of both smoking and alcohol drinking are essential. Further studies to clarify the role of green tea, consumed at both high and low temperature, in the prevention of esophageal cancer would also be valuable.
